# Empowering learners through student-led integration of environmental health into small group discussions

**DOI:** 10.1080/10872981.2025.2534054

**Published:** 2025-07-27

**Authors:** Eunheh Koh, Joyce Kim, Fatma Aldihri, Hannah Huang, Michael Murray, Nicole Winston, Christopher M. Watson

**Affiliations:** a Department of Academic Affairs, Medical College of Georgia at Augusta University, Augusta, Georgia; b Department of Biological Sciences, and Institute of Public and Preventive Health, Augusta University, Augusta, Georgia; c Department of Pharmacology and Toxicology, Medical College of Georgia at Augusta University, Augusta, Georgia; d Department of Pediatrics, Medical College of Georgia at Augusta University, Augusta, Georgia

**Keywords:** Environmental health curriculum, small group discussions, student-led, case-based learning, public health education

## Abstract

With ongoing climate change and other major human-induced changes to the biosphere, there is a greater need to improve future healthcare providers’ environmental health (EH) literacy. As of 2022, 45% of U.S. MD programs lacked a required EH curriculum. A self-assembled group of four medical students conceptualized and planned this pilot study to characterize matriculating medical students’ EH knowledge and attitudes. This group also developed EH content for integration into a preexisting 18-month Case-Based Learning (CBL) curriculum to enhance small-group discussion and learning. Matriculating medical students were invited to participate in an anonymous cross-sectional survey assessing EH literacy and the need for an EH-specific curriculum in August 2023. Concurrently, the student group analyzed 44 cases in the current CBL curriculum and searched PubMed and the PEHSU Climate Resources for Health Education for pertinent topics from the case review. The group then formulated learning objectives and discussion questions for the facilitator guide for 30 cases, with expert review by curriculum faculty members. 70 of 200 students (35%) fully completed a survey about EH literacy. Eighty percent of students reported no previous coursework pertinent to EH, with most students demonstrating a basic understanding of the concept. Students reported low confidence in counseling patients regarding pertinent EH matters and a limited understanding of social determinants of health pertinent to the local area. In 30 identified medical conditions across 10 disciplines, 57 new objectives were developed to address environmental exposures, infectious diseases, climate change, and local implications. Increasing EH literacy among medical students represents a high-impact educational need. This pilot study, conceived and led by medical students, successfully characterized the EH knowledge gap among medical students and integrated novel discipline-specific learning objectives and discussion points into a pre-existing CBL curriculum. This model may easily be adapted to other institutions’ curricula.

## Background

**Figure 1.**
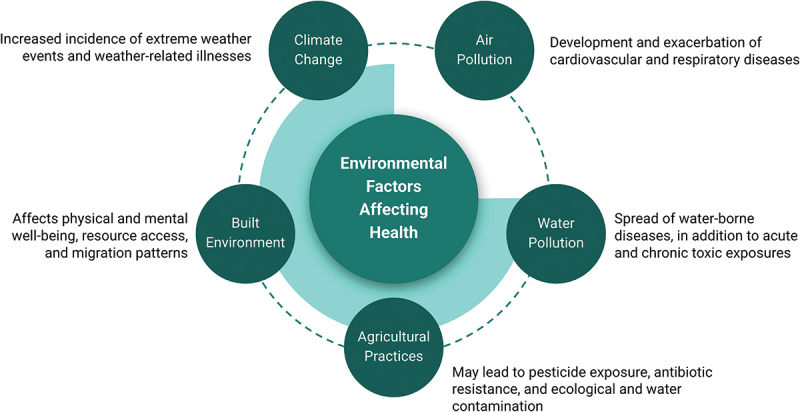
A brief overview of various environmental factors and their impacts on human health.

Environmental health (EH) studies the effects of the environment on human health [1,2]. Environmental factors, including air pollution and climate change, contribute to significant morbidity and mortality worldwide [2–5] (Figure 1). Poor health outcomes associated with climate change disproportionately affect communities of color and low income [6], requiring health providers to have a strong understanding of EH to address these disparities.

This is an adaptation of an original work: [How the environment impacts our health]. Pan American Health Organization/World Health Organization (WHO); [2019]. License: CC BY-NC-SA 3.0 IGO. This adaptation was not created by WHO. WHO is not responsible for the content or accuracy of this adaptation. The original edition shall be the binding and authentic edition.

Nationally, medical school curricula have a lack of EH teaching. According to a report from the Association of American Medical Colleges, 45% of US M.D. medical schools did not have an EH curriculum in 2022 [7]. Additionally, research has documented a need among healthcare providers for training to understand and manage environmental exposures [8,9]. EH education can improve the environmental health literacy (EHL) and self-efficacy of future providers in providing guidance to patients on ways to reduce EH risks.

**Figure 2.**
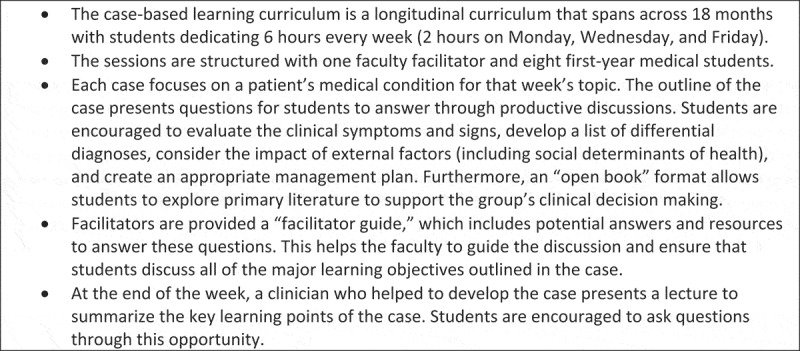
The structure of the case-based learning curriculum (CBL) at the Medical College of Georgia.

Building on previous studies reporting improved student understanding with additional EH education [10,11], a group of four medical students with an interest in public health integrated EH content into the pre-existing curriculum at the Medical College of Georgia. Case-based learning (CBL) has improved knowledge retention and communication skills [12] and was highlighted for its efficacy in another pilot EH curriculum [11]. Thus, the longitudinal CBL curriculum represented an ideal opportunity for EH content integration. Students discuss one clinical case weekly under a faculty facilitator’s guidance, which provides a comprehensive overview (Figure 2).

Supplemental EH education through CBL provides an opportunity to highlight the health effects of environmental exposures and build the necessary communication skills to discuss risks with patients. CBL offers a feasible, replicable, and time-efficient mechanism for adapting the curriculum to incorporate EH.

This study aimed to elucidate gaps in student knowledge of EH, develop new EH content for small-group learning, and demonstrate an approach to integrating this content into a pre-existing CBL curriculum that can be broadly applicable to other institutions.

## Understanding baseline knowledge gaps

**Figure 3.**
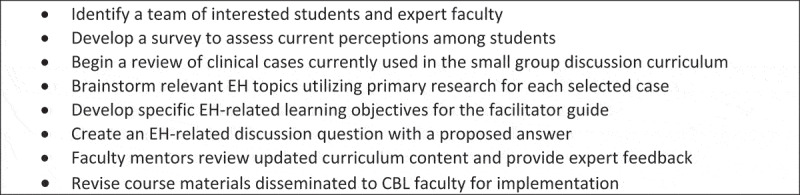
Curriculum review process for environmental health content development and integration.

A systematic curriculum review cycle was followed to identify and address the knowledge gaps (Figure 3). As part of this process, an anonymous cross-sectional survey was administered to first-year students in August 2023. The inclusion criteria included first-year medical students who voluntarily consented to the study. Anyone who did not meet these criteria was excluded from this study. Students were recruited by email. The study was approved by the Augusta University Institutional Review Board and received an exempt status. Students provided demographic information and described their baseline knowledge surrounding EH. Likert scale questions assessed the participants’ perceptions of five general statements about EH. Manifest content analysis identified themes in open-ended responses [13]. Total counts and percentages were calculated for the EH themes surveyed on a 5-point Likert scale.

Overall, 91 surveys (45.5%) were collected during recruitment; 70 complete responses (35%) were collected. The surveyed students were, on average, 23 years old and predominantly female, with a response rate of 64% female and 36% male, reflecting class demographics [14]. The extent of previous EH exposure varied, with 16% noting enrollment in undergraduate courses, 1% in graduate coursework, and 3% reporting EH research experience. Eighty percent of students did not have any previous EH exposure. Content analysis identified themes when students described their ‘current understanding of environmental health’ (Table 1). Overall, students demonstrated a rudimentary understanding of EH. Students were asked to rate their ability to counsel patients on EH topics using statements from a previous study [10] (Table 2). Only 24.3% and 27.1% of students expressed confidence in counseling patients about pollutants and the effects of climate change on health, respectively. Students’ self-reported knowledge about the impact of social determinants of health (SDOH) on local, national, and global scales was also assessed. Forty-two percent of students reported a limited understanding of SDOH in our local community in Augusta, Georgia (Table 2). This deficit became a priority for the proposed curriculum updates, aiming to enhance students’ understanding and clinical competence during the clerkship curriculum based in this city.

**Table 1.** Self-reported current understanding of environmental health.

	Frequency (%)	Selected Quotes
Limited Knowledge	21.4	‘I do not understand it very well at all.’ ‘I do not have much of an understanding of the environment aside from the ecology class I’ve taken.’
Effects of the Environment on Human Health	52.9	‘How environmental factors in the environment (including air quality, water quality, environmental toxins, climate change, pollution, etc.) affect health outcomes.’ ‘Environmental factors also contribute to health outcomes. For example, growing up in an area with poor drinking water or an area in a food desert are environmental factors that contribute to individuals’ health.’ ‘I understand that taking in environmental factors is important to take into consideration while taking a patient history.’
Effect on the Environment	7.1	‘I understand that environmental health is the study of how the environment is taken care of and how we can better sustain the environment in a positive manner.’ ‘Many human/societal activities are performed with disregard to the environment causing imbalance and harm to all that are connected to said environment.’
Bidirectional Relationship between the Environment and Health	14.3	‘It is the relationship that occurs between people and the environment, with effects occurring both ways.’ ‘Environmental health is a branch of public health that involves how environmental factors and the health of the environment affect people and the population.’
Environmental Health and Climate Change	4.3	‘I understand that the current climate changes have led to overheating, extreme weather patterns, and a more rapid depletion of natural resources. This is leading to worse health outcomes, wild animal displacements, and an overall impact on the health of the world.’ ‘Environmental health will play a major role in human health within the next few years due to climate change and increases in dangerous gases into the environment.’

**Table 2.** Student agreement levels on likert scale (1 = strongly agree, 5 = strongly disagree). Lower average scores indicate stronger agreement, while higher average scores indicate stronger disagreement.

Statement	Mean	Standard Deviation
‘I know enough to advocate in my community to try to reduce the health-related impact of environmental toxins on air and water quality.’	3.4	1.01
‘I feel prepared to discuss the specific impacts of climate change on human health.’	3.2	1.07
‘I have a good understanding of the social determinants of health that are prevalent in the Augusta community.’	3.1	0.99
‘I have a good understanding of the social determinants of health that play a role in national health disparities.’	2.2	0.77
‘I have a good understanding of the social determinants of health that play a role in global health disparities.’	2.4	0.93

## EH curricular integration into small group discussions

After reviewing all cases in the current curriculum, 30 medical conditions were selected. Several cases had multiple weeks for discussion, including diabetes and pregnancy. Along with a discussion question and answer for each case, 57 new learning objectives about environmental exposures, environmental justice, climate change impacts, and patient counseling were developed (Table 3). A literature review and review by faculty ensured relevance of the proposed objectives. With greater awareness of environmental risk factors, students may be more likely to discuss pollutants’ impacts with patients and counsel them on minimizing exposures. Through the environmental justice component of the curriculum, students may have a greater capacity to advocate for communities experiencing a higher environmental burden and consider EH’s intersectionality to other SDOH [6]. Climate change topics, such as increased spread of vector-borne diseases and heat waves, were included to prepare students for emerging health challenges. Additional topics may be covered based on the groups’ discussions.

**Table 3.** Environmental health objectives by discipline and disease process.

Musculoskeletal	
Rheumatoid Arthritis (RA)	Identify various environmental risk factors that have been associated with the pathogenesis of RA, including smoking and air pollution
**Endocrine**	
Diabetes	Recognize environmental factors that may contribute to the worsening severity of diabetic disease, including air pollution, polyfluoroalkyl substances (PFAS), and insufficient green spaces Discuss the impact of food deserts on diabetes prevalence Use the Food Research Atlas to explore the factors that influence food insecurity (such as access to supermarkets and vehicles) in our surrounding community
Thyroid Cancer	Elaborate on how exposure to ionizing radiation is associated with papillary thyroid carcinoma Consider how the proximity of the patient’s home to a local nuclear power plant in Augusta could present a risk if there were an accident Identify important environmental health questions to ask when isolating a differential diagnosis
**Cardiovascular**	
Myocardial Infarction (MI)	Explore the environmental risk factors that may affect one’s risk of developing a STEMI, including heat exposure and air pollution
Congestive Heart Failure (CHF)	Identify specific air pollutants that have been correlated with the pathogenesis and morbidity and mortality from CHF Characterize the air pollution burden in the Augusta area and who may be more vulnerable to exposure Discuss the disproportionate burden of air pollution that may affect minority communities secondary to proximity to major roadways
Hypertension	Discuss the impact of medications, such as diuretics, on altering one’s heat tolerance Identify populations that are more susceptible to heat stress, such as elderly individuals who may have impaired thermoregulatory capacities or individuals with outdoor occupations Describe how to counsel patients vulnerable to extreme heat and its importance with climate change exacerbated heat waves
Congenital Heart Disease	Identify various environmental risk factors for congenital heart disease, including heavy metal exposure and gaseous pollutants Consider how these environmental burdens may lead to disparate outcomes worldwide
**Pulmonary**	
Chronic Obstructive Pulmonary Disease (COPD)	Discuss the impact of temperature and air pollution on the risk of infection in patients with COPD Summarize how one could counsel a patient to prevent future exacerbations, such as using resources like the Air Quality Index
Cystic Fibrosis (CF)	Determine how increasing ambient temperatures in the setting of climate change and air pollution may increase the risk for infection in patients with CF
Evaluation of Cough	Evaluate the impact of occupational exposures to silica, carbon dust, beryllium, and asbestos Identify sources of indoor and outdoor air pollution that may contribute to cough, including mold
House Fires	Establish potential sources and toxins found in smoke in the setting of a house fire
**Gastrointestinal**	
Cirrhosis	Discuss three mechanisms of how individuals can be exposed to toxins in our environment (ingestion, inhalation, transdermal absorption) Identify causes of cirrhosis, including the impact of organochlorine pesticide exposure Recognize how to counsel patients who frequently use pesticides and reduce their exposure (such as wearing the appropriate personal protective equipment and changing clothes after work)
Hemochromatosis	Understand how environmental factors may exacerbate a genetic disease like hemochromatosis Consider obtaining a detailed diet history to identify increased iron consumption
**Genitourinary**	
Nephrolithiasis	Understand the mechanism of heat exposure and subsequent dehydration can lead to increased risk of nephrolithiasis Discuss the concept of ‘redlining’ and its impacts of exacerbating environmental burdens in certain communities, specifically to the urban heat island effect
Chronic Kidney Disease	Explore environmental factors that may lead to the pathogenesis of chronic kidney disease, including toxins, heavy metals, and air pollutants
Prostatic Adenocarcinoma	Identify environmental exposures that have been associated with the pathogenesis of prostatic adenocarcinoma
**Hematology**	
Sickle Cell Disease	Describe what environmental factors (such as air pollution or temperature changes) may contribute to sickle cell-related vaso-occlusive crises. Consider how to counsel a patient with sickle cell disease to avoid certain factors that trigger acute exacerbations.
Leukemia	Identify environmental risk factors that may increase one’s risk for developing hematologic cancers, including alkylating agents, pesticides, and radiation
Venous Thrombosis (VTE)	Explore the current evidence in regard to air pollution on the coagulation cascade and its implications
**Neurology**	
Learning Disorders	Investigate the impact of various toxic exposures (such as lead and mercury) that have been associated with neurodevelopmental outcomes
Idiopathic Intracranial Hypertension (IIH)	Identify obesity as a risk factor for IIH Assess how climate change may affect the prevalence of obesity, including impaired production of produce and fewer opportunities for exercise with extreme weather events
Parkinson’s Disease	Discuss occupational and environmental risk factors, including organophosphate pesticides, that may lead to new or worsening Parkinson’s disease
Trigeminal Neuralgia	Identify that occupational exposure to paint removers (due to trichloroethylene and its metabolite) may trigger Trigeminal Neuralgia
Multiple Sclerosis	Summarize some of the lifestyle modifications important to discuss with a patient who is newly diagnosed with MS related to heat, particularly in the setting of anthropogenic climate change
Febrile Seizure	Examine the risk factors that can lead to febrile seizures Discuss other environmental toxicants and how they may contribute to the risk of seizures
Lead Poisoning	Identify clinical signs and risk factors that raise suspicion for lead poisoning Recognize risk factors for childhood lead exposure, including homes built earlier than 1978, occupational exposures, and imported toys Characterize the high prevalence of lead poisoning in our local community and identify local resources available (Department of Public Health, Lead Hazard Reduction Program)
**Maternal & Fetal Health**	
Breast Cancer	Characterize various environmental exposures associated with an increased risk of breast cancer, including exposure to dichlorodiphenyltrichloroethane (DDT) and chemicals in certain cosmetic products. Further, discuss how patients may be exposed to these chemicals with the lipophilic and pervasive nature of DDT in our environment. Discuss the concept of environmental justice and how certain communities may be more vulnerable to chemical exposure based on the neighborhood they live in and the proximity to toxic chemical sites Consider how patients may prevent extensive exposure to certain chemicals, including available resources in the community
Pregnancy	Understand the impacts of prenatal exposure to certain chemicals, including PBDEs (flame retardants), lead, air pollution, and secondhand smoke Recognize that certain chemicals can be passed down from mother to baby due to their lipophilic nature and may pose an increased risk for the fetus Discuss how you would counsel a pregnant patient on how she may minimize exposure to toxicants Identify the risk factors for oligohydramnios, particularly in relation to maternal dehydration and subsequently increased risk with climate change
**Infectious Diseases**	
Lyme Disease	Explore the impacts of climate change on tick-borne diseases, such as the changing temperature in the spread of disease in non-endemic areas Consider how this phenomenon may also affect other vector-borne diseases
Varicella Pneumonia	Discuss the impact of environmental factors such as air pollutants on varicella pneumonia Explore the greater implications of climate change on airborne diseases

The student group also identified applications of EH in Augusta to help students build counseling skills for clinical rotations. As Augusta has some of the highest childhood lead exposure statewide [15], the group emphasized the clinical signs, diagnosis, and associated health outcomes. Resources from the local Department of Public Health were shared so students could help with the referral process. Local industrial risks, including chemical factories and a nuclear power plant, were highlighted, encouraging students to research pertinent hazards. This local focus equips students with the knowledge to counsel patients and fosters advocacy for vulnerable populations.

## Conclusion

This student-conceptualized and led pilot study highlights the need for an EH-focused curriculum to enhance medical students’ EHL and the feasibility of developing and deploying this content through CBL. This model is a practical way for medical students to lead similar initiatives by identifying a method to integrate EH topics into a pre-existing discussion-based curriculum. Furthermore, it offers the opportunity to focus on local environmental issues, strengthening both EHL and advocacy efforts. Incorporating EH topics early in medical education helps students understand the impact of environmental factors and SDOH on patient health, empowering them to become better advocates during clinical rotations.
